# hCG, the wonder of today's science

**DOI:** 10.1186/1477-7827-10-24

**Published:** 2012-03-28

**Authors:** Laurence A Cole

**Affiliations:** 1 USA hCG Reference Service, Albuquerque NM 87104, USA

## Abstract

**Background:**

hCG is a wonder. Firstly, because hCG is such an extreme molecule. hCG is the most acidic glycoprotein containing the highest proportion of sugars. Secondly, hCG exists in 5 common forms. Finally, it has so many functions ranging from control of human pregnancy to human cancer. This review examines these molecules in detail.

**Content:**

These 5 molecules, hCG, sulfated hCG, hyperglycosylated hCG, hCG free beta and hyperglycosylated free beta are produced by placental syncytiotrophoblast cells and pituitary gonadotrope cells (group 1), and by placental cytotrophoblast cells and human malignancies (group 2). Group 1 molecules are both hormones that act on the hCG/LH receptor. These molecules are central to human menstrual cycle and human pregnancy. Group 2 molecules are autocrines, that act by antagonizing a TGF beta receptor. These molecules are critical to all advanced malignancies.

**Conclusions:**

The hCG groups are molecules critical to both the molecules of pregnancy or human life, and to the advancement of cancer, or human death.

## Background

Let's get to the point, why do we call human chorionic gonadotropin (hCG) the wonder of today's science. Firstly, hCG is an extreme molecule. It is the most acid protein in humans, some hCG variants have a peak isoelectric point (pI) stretching to pI 3.1. hCG variants are the most sialylated glycoproteins with up to 15 sialic acid residues per molecule. hCG variants are the most glycosylated of glycoproteins, hCG containing 30% sugar by molecular weight, hyperglycosylated hCG containing 39% sugar and hyperglycosylated hCG free ß-subunit containing 42% sugar by molecular weight. Finally, with its extreme molecular weights, hCG is the longest circulating molecule in human blood with a circulating half life of 36 hours. Secondly, as described in this review, there are amazingly 5 unique variants of hCG, each having identical amino acid sequence, produced by different cells and having independent functions. These are hCG, sulfated hCG, hyperglycosylated hCG, hCG free ß-subunit and hyperglycosylated hCG free ß-subunit. There is no other molecule like hCG.

Finally, hCG and its variants have an incredibly wide spectrum of biological functions. These range from hyperglycosylated hCG and pregnancy implantation and placental development, to hyperglycosylated hCG and hCG and hemochorial placentation. They also include hCG and fetal and uterine growth and numerous other key functions during pregnancy. Sulfated hCG is produced by the pituitary in women and controls steroidogenesis during the menstrual cycle and ovulation of the oocyte. Fascinatingly, a hyperglycosylated hCG/hCG free ß pathway is the center-point of all advanced human cancer biology, driving cancer growth, cancer invasion and cancer malignancy. This is not forgetting the key evolutionary role that chorionic gonadotropin variants play in the evolution of humans, most notably development of the hemochorial placentation system, that supports the development of the human brain. You could call hCG and its variants the everything molecules.

A common question is why are the 5 independent variants of hCG all called hCG. This is because they all share a common α-subunit and ß-subunit amino acid sequence.

I remember when I first discovered hyperglycosylated hCG as an hCG variant back in 1977 [1]. I first named it "invasive trophoblast antigen" because we knew at that time that it independently drove implantation of pregnancy and invasion by choriocarcinoma cells. Within 2 years I received an official letter from the World Health Organization instructing me to rename it a molecule containing the name hCG, because it contains hCG amino acid sequence. I renamed it hyperglycosylated hCG based on its structure.

**Figure 1:**
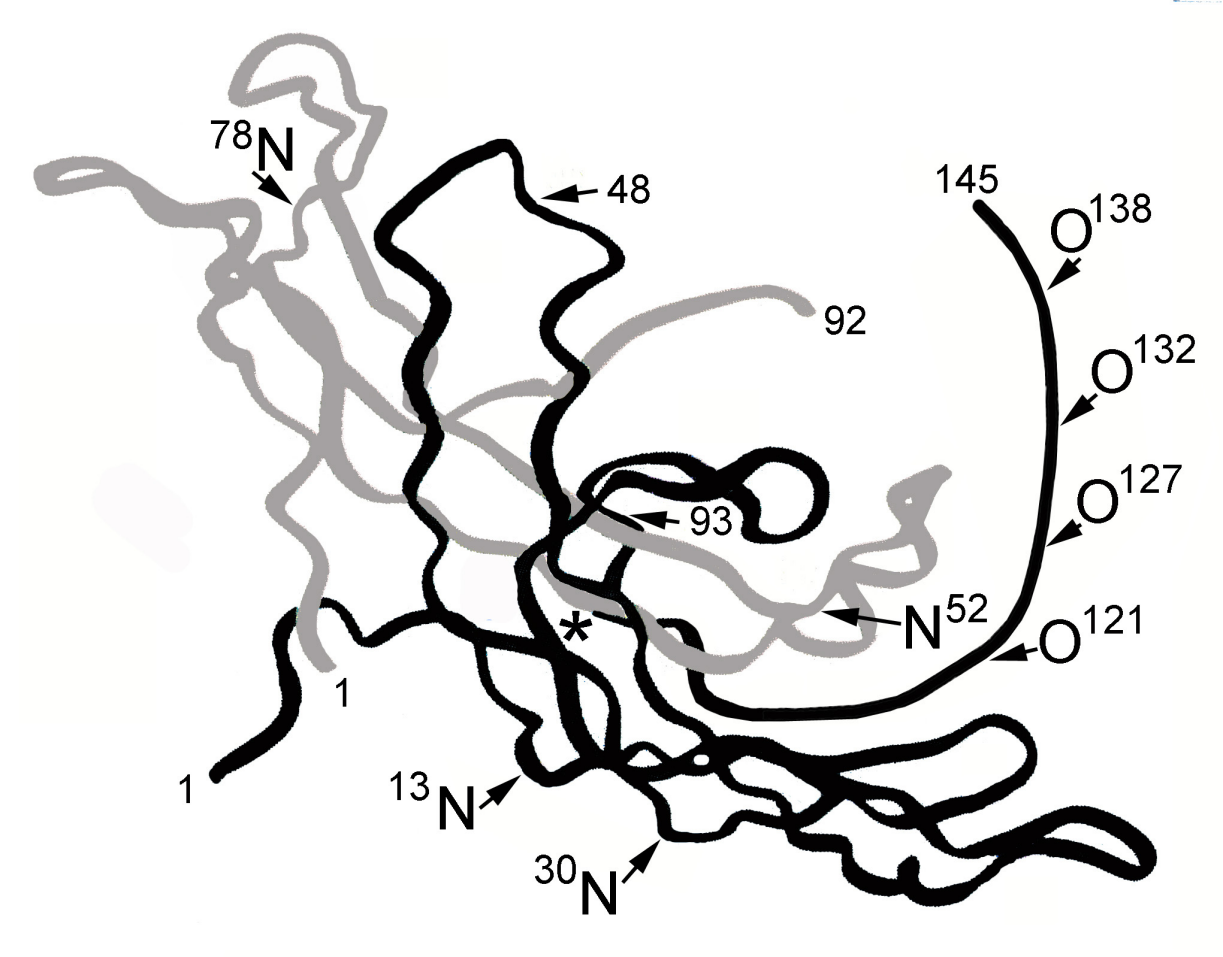
**The three dimensional structure of deglycosylated hCG as shown by X-ray defraction **[4]. O and N mark O-linked and N-linked oligosaccharides and * marks the site of the cystine knot, common to TGFß. Grey is α- and black is ß-subunit.

Research by Laub and Jennissen [2] and Lehnert and Akhurst [3] showed that hCG ß-subunit was part of the transforming growth factor ß (TGFß) oncoprotein family of molecules. Lapthorn and collegues determined the 3 dimension structure of the hormone hCG [4], and showed a 4 peptide cystine knot structure in the ß-subunit was common to TGFß (Figure 1). It is well established that the hormone hCG produced in pregnancy [5] and the sulfated variant of hCG produced by the pituitary [6], act on an hCG/luteinizing hormone (LH) receptor to evoke a response. Interestingly, hyperglycosylated hCG and hCG free ß-subunit have been shown to be autocrines and to function separately, binding and antagonizing a TGFß receptor on the cells that produce these hCG forms [7,8]. So another wonder, hCG and its variants have two very different receptor binding sites. Here we examine hCG and it variants, the wonders of today's science.

### hCG, hyperglycosylated hCG, hemochorial placentation and evolution

A major function of hCG during pregnancy can be described as driving hemochorial placentation, or the efficient method whereby humans drive nutrient transfer to the fetus. hCG, fetal hCG and hyperglycosylated hCG seeming have many critical roles during pregnancy (See Section hCG, hyperglycosylated hCG and pregnancy). Almost every medical text book sold today describe the sole function of hCG as driving luteal steroidogenesis. This is a very out of date description of hCG, summarizing research in the 1910s, 1920s and 1930s [9-12], books surely must be more up to date than this period. Over 100 publications in the 1970s-2010s describe and confirm the many established functions of hCG variants. Why is everything so out of date? Looking at the section of this review on hCG function during pregnancy, if there is only room to describe just one of the many functions it should be driving hemochorial placentation as described here, and not just maintaining progesterone production by the corpus luteum for 3-4 weeks.

**Figure 2:**
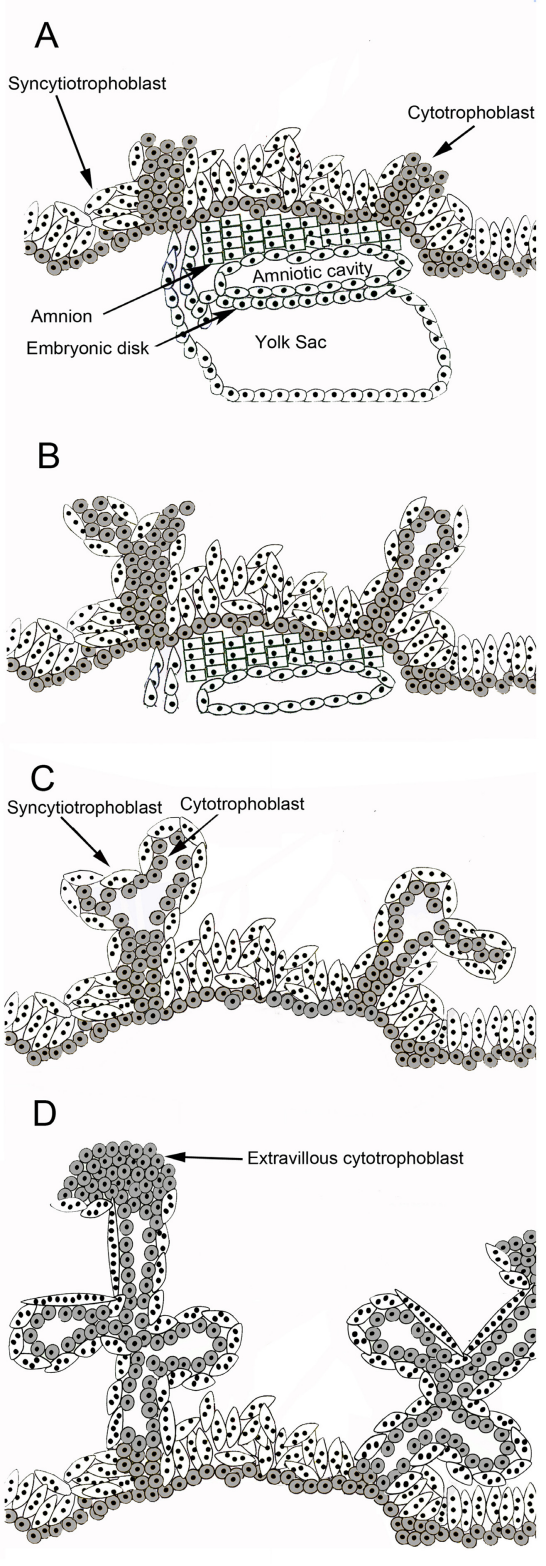
**Formation of villous trophoblast**. **A**. Cytotrophoblast columns in early implanted embryo. B. Extension of columns and differentiation of peripheral cells. **B**. and **C**. Folding of extensions caused by shape of syncytiotrophoblast cells. **C **and **D **formation of trophoblastic villi. No vascular supply, spiral arteries or fetal vasculature is shown.

It is well established that hyperglycosylated hCG drives invasion and implantation by placental trophoblastic cell deep into the myometrium of the uterus [13-16]. Hyperglycosylated hCG drives cytotrophoblast cell growth [8,13,14,16,17], and hCG promotes the fusion and differentiation of peripheral cytotrophoblast cells, where the blood supply is, to syncytiotrophoblast cells [17,18]. Hyperglycosylated hCG and hCG lead the implantation of placenta tissue into the uterus and the formation of villous trophoblast tissue. As illustrated in Figure 2 panel A, implanted blastocysts form columns of cytotrophoblast cells. Columns extend under the influence of hyperglycosylated hCG. As illustrated in panels B and C, hCG promotes differentiation of peripheral cells to active syncytiotrophoblast cells, closest to the circulation. Shape of syncytiotrophoblast cells forces arm formation and folding in developing villi (Figure 2 panels C and D). Taken together this generates villous trophoblastic tissue (panel D).

**Figure 3:**
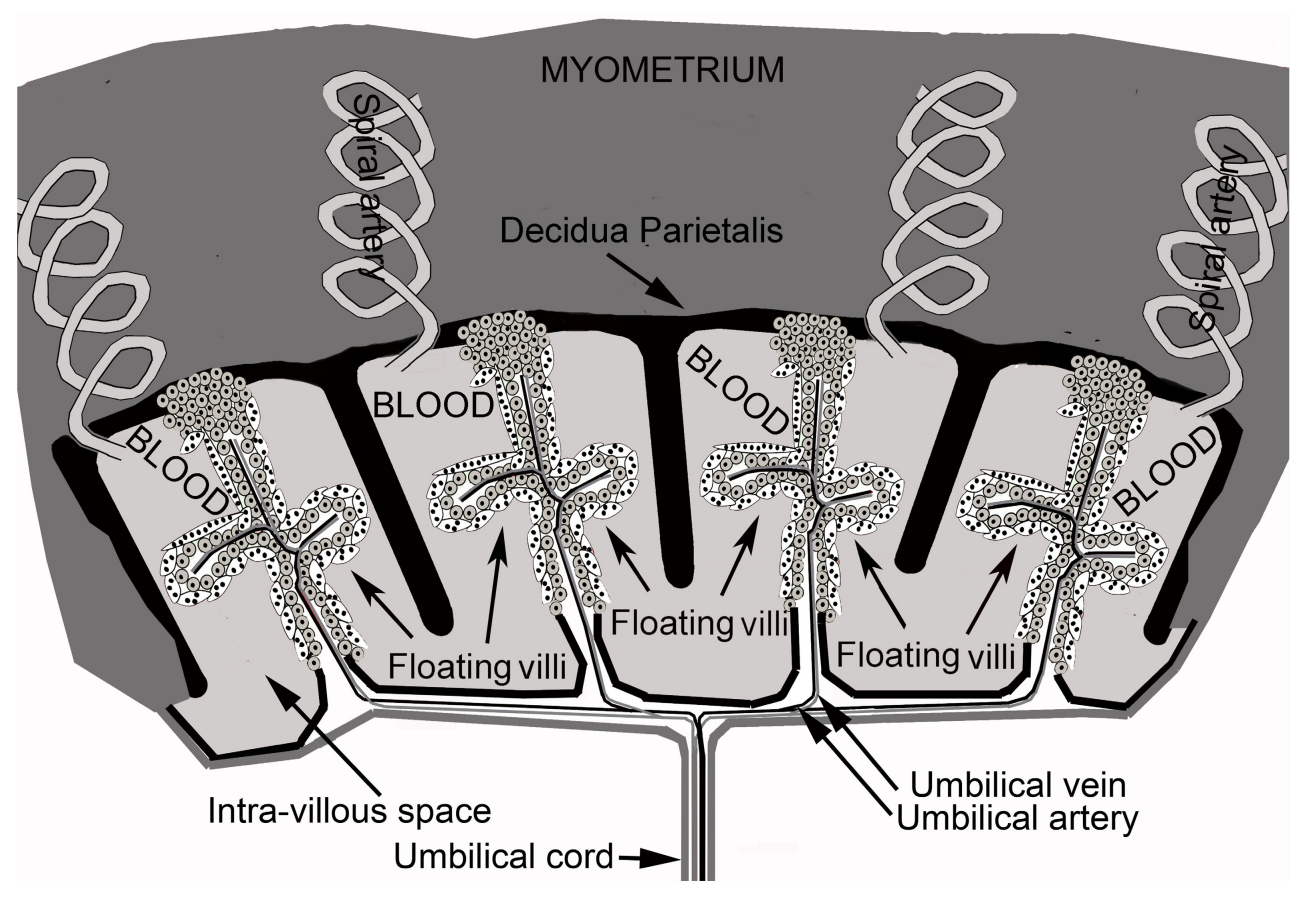
**Human placental hemochorial placentation**. While hCG and hyperglycosylated hCG force villous trophoblast tissue formation [13-17], hCG promotes the development and growth of uterine spiral arteries [19-26]. Angiogenesis forces the protrusion of arteries to reach invading villous trophoblast tissue [19-26]. hCG also promotes the formation of the umbilical circulation in villous tissue and the formation of the umbilical cord [27-32]. All linked together, villous trophoblast tissue, maternal spiral artery blood and fetal umbilical circulation and you have hemochorial placentation, efficient fetal nutrient exchange, as illustrated. In hemochorial placentation, spiral artery bring maternal blood into one of 4-7 hemochorial placentation chambers. Blood fills the chamber, nutrients (oxygen/glucse/amino acids) them pass across syncytiotrophoblast cells into villous side-arms or floating villi. They are then rapidly absorbed by the umbilical circulation.

While hCG and hyperglycosylated hCG force villous trophoblast tissue formation, hCG promotes the development and growth of uterine spiral arteries [19-26]. Angiogenesis forces the protrusion of arteries to reach invading villous trophoblast tissue [19-26]. hCG also promotes the formation of the umbilical circulation in villous tissue and the formation of the umbilical cord [27-32]. While there is no clear evidence of how placental villi, the maternal uterine spiral arteries and fetal umbilical circulation are tied together to activate hemochorial placentation, all the component of hemochorial placentation are clearly hCG and hyperglycosylated hCG controlled. Figure 3 shows a human placenta and active hemochorial placentation. Histology shows that hemochorial placentation only becomes active by 10 weeks gestation. As illustrated in Figure 3, maternal blood fills the decidua parietalis chambers. Nutrients are passaged accross syncytiotrophoblast cells and into placental villi and into the developed fetal umbilical circulation.

Hyperglycosylated hCG functions in implantation are proven [13-16]. That hyperglycosylated hCG drives cytotrophoblast growth is shown and confirmed [8,13,14,16,17]. That hCG promotes the fusion of peripheral cytotrophoblast cells to syncytiotrophoblast cells is also proven [18]. That hCG drive uterine artery angiogenesis is demonstrated and confirmed multiple times [19-26]. Finally that hCG forms the umbilical circulation has been demonstrated [27-32]. Putting all these synthetic facts together, putting villous trophoblast tissue with maternal spiral arteries with fetal uterine circulation and you have hemochorial placentation. Clearly, the combination of hCG and hyperglycosylated hCG drive all event leading to hemochorial placentation [14]. This is suggested, however, but it has not been proven. As described in Section hCG, Hyperglycosylated hCG, Hemochorial Placentation and Evolution, human hCG is dramatically different to primate hCG. Can we prove that hCG and hyperglycosylated hCG drive hemochorial placentation in humans. No, the research would be unethical. The only evidence that hCG related molecules are the driving force of hemochorial placentation is the first appearance of invasive hemochorial placentation in early simian primates. Primates first evolved 80 million years ago, early simian primates evolved just 37 million years. Interestingly, chorionic gonadotropin (CG) and hyperglycosylated CG first evolved in this same species. The logical reason that hemochorial placentation developed is the parallel evolution of its driving signal CG and hyperglycosylated CG.

Assuming that CG and hyperglycosylated CG drive hemochorial placentation during pregnancy (see Section hCG, Hyperglycosylated hCG, Hemochorial Placentation and Evolution) we examine human and primate models (Table 1). Table 1 quotes numerous evolution publications [33-44]. As illustrated, prosimian primates, example: lemur, produced LH in pregnancy, biopotency 1X. This primate used inefficient non-implanting epitheliochorial placentation to manage pregnancy. This primate had a tiny brain, only 0.07% (^1^/_1428 _th) of body weight. With more advanced early-simian primates, example: old world monkey, evolved CG from a deletion mutation in LH. CG was produced by fused syncytiotrophoblast cells and hyperglycosylated CG was produced by cytotrophoblast stem cells [7,45]. With the evolution of CG and hyperglycosylated CG the early-simian primate developed hemochorial placentation (Table 1). The initial CG produced by this species was deficient in acidity with only 5 acidic oligosaccharides. It has only a 2.4 h calculated circulating 1/2-life (Table 1) or had only 7.3X greater potency than prosimian primate placental LH. Early simian hyperglycosylated CG had only the potency to implant placenta through the thickness of the uterine inner lining or decidua. Hemochorial placentation in early-simian primate was barely more efficient than placentation in prosimian primates, leading to the development of a brain 0.17% (^1^/_588 _th) of body mass (Table 1) [33-44].

**Table 1:** Parallelisms between placental implantation and hemochorial placentation in primates, sugar structure on CG or LH, and relative brain masses

Species	Placentation characteristics	Depth of Invasion	Molecule produce; # oligosaccharides (oligos); pI of dimer; circulating 1/2-life; relative biopotency	Brain mass(% body weight)	First appearance (million years ago)
Humans	Hemochorial	1/3 rd myometrium	CG; 8 oligos; pI 3.5; 1/2-life 36 h, 109X	2.4%	0.1

Advanced simian primates	Hemochorial	1/10th myometrium	CG; 6 oligos; pI 4.9; 1/2-life 6 h; 18X	0.74%	20

Early simian primates	Hemochorial	through decidua	CG; 5 oligos; pI 6.3; 1/2-life 2.4 h; 7.3X	0.17%	37

Prosimian primate	Epitheliochorial	non-implanting	LH; 3 oligos; pI 9.0; 1/2-life 0.33 h; 1X	0.07%	55

The circulating 1/2-life of Advanced simian primate and Early simian primate CG was calculated using an equation that consider number of oligosaccharides on LH and human CG and the known circulating 1/2 life. C1/2 is circulating 1/2-life and number of oligosaccharides is *#O*, *CR *= 2.4*^#O ^*x 1.9. Table summarizes published data (34-45)

With advancing evolution came advanced simian primates, example: orangutan. Following mutation in the CG genes, advanced simian primates produced a more acidic CG with 6 acidic oligosaccharides. This had a calculated circulating 1/2-life of 6 hours (Table 1) or had a biopotency of 18X more than prosimian LH. With it extra biopotency hyperglycosylated CG implanted this placenta through the decidua and to 10% of the width of the uterine wall or myometrial muscle. This species had significantly more potent hemochorial placentation leading to the development of a brain of 0.74% of body mass (Table 1) [33-44]. Humans evolved after many mutations in the hCG genes. This led to a super acidic hCG, pI 3.5 with 8 acidic oligosaccharides. This human hCG is the most acidic glycoprotein with the highest proportion of sugars occurring in any primate or in humans. This super-hCG raised the circulating 1/2-life to an incredible 36 hours, or to a biopotency of 109X over prosimian LH. It is this super hCG and super-hyperglycosylated hCG that drove implantation to as deep as 1/3^rd ^the thickness of the myometrium (Table 1), and drove hemochorial placentation to the extreme of efficiency. It was this super-hCG and this hemochorial placentation that permitted the development of the 2.4% of body mass human brain.

The mechanism whereby humans developed ultra-efficient placentation has defied evolutionary scientist for many years [37-39]. This is all explained by the evolution of CG (biopotency 7, 3X), and the advancing evolution of CG, early-simian primate (biopotency 18X), advanced simian primate (18X) and humans (109X). In the human fetus, developing the large brain is not easy, it uses 60% of transferred glucose and oxygen, leaving the development of some human organs lacking in nutrients [37-39].

In many respects this review of the role of CG and hyperglycosylated CG in human evolution does not end at this point. Recent research shows that these potent implantation and growth factors are at the root of pregnancy failures in humans (see Section Hyperglycosylated hCG, Failing Pregnancy and Hypertense Pregnancy). Also human malignancies take advantage of these super-potent invasion and growth factors in the human genome to stimulate growth and invasion of all advanced human malignancies. This is discussed in Section Hyperglycosylated hCG, hCG free ß-subunit and Cancer. CG is a wondrous molecule that seemingly was only generated to advance evolution to humans. Unfortunately humans have to live with the terrors of this wondrous molecule in their genome.

### hCG, hyperglycosylated hCG and pregnancy

**Figure 4:**
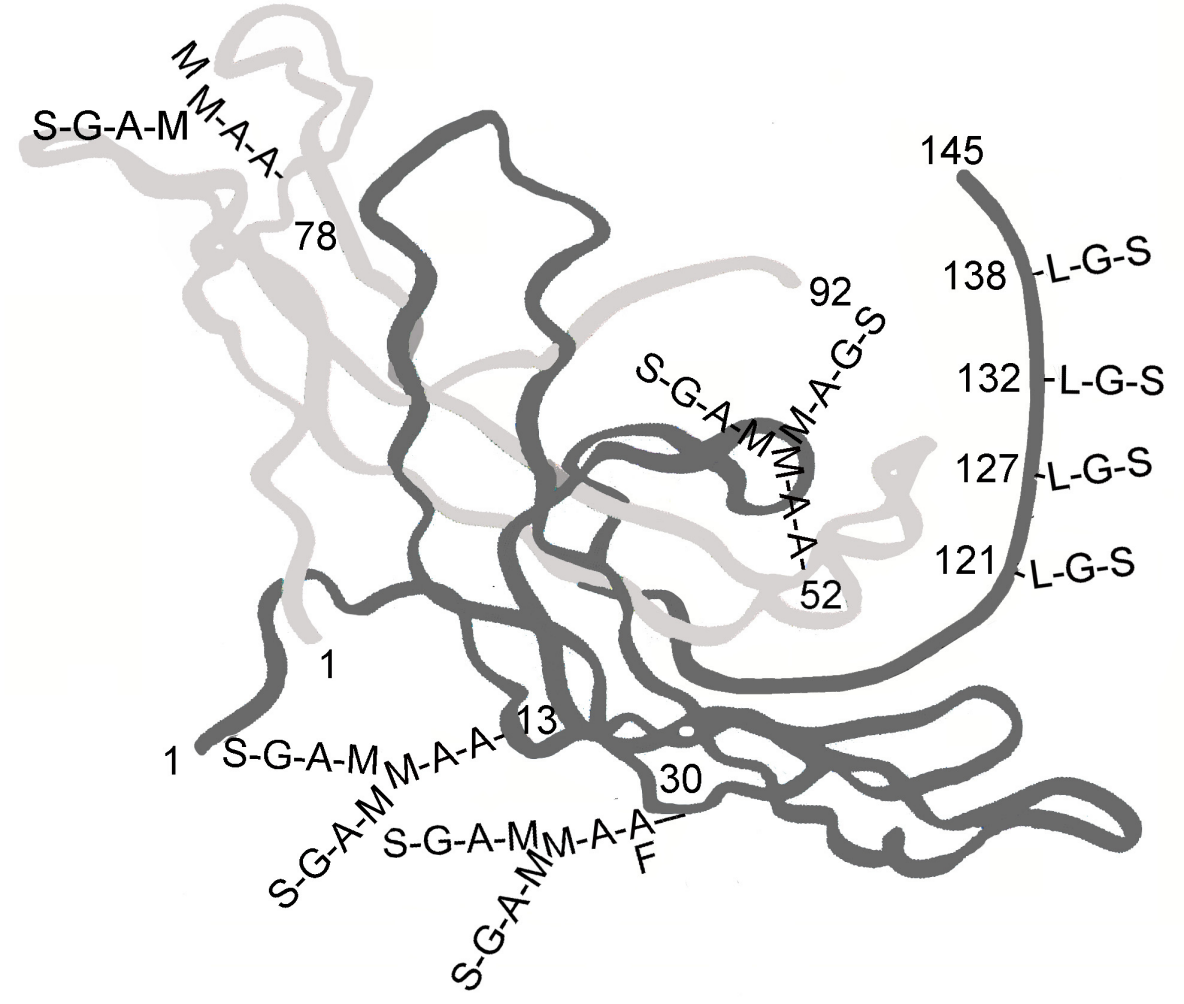
**The three dimensional structure of hCG **[4]. In order to form crystals hCG was first deglycosylated. hCG and hyperglycosylated hCG have identical amino acid sequence [1], yet vary in function. hCG binds the hCG/LH receptor while hyperglycosylated hCG antagonizes a TGFß receptor [6-8]. As such the glycosylation (all removed) has to make a structural difference to the two hCG forms. This difference is not shown. Oligosaccharide are added to the three dimensional structure assuming the carbohydrate structure observation of Elliott et al. [1]. Oligosaccharides are charged so must project towards the surface of the molecule. L in oligosaccharides is N-acetylgalactosamine, A is N-acetylglucosamine, S is sialic acid or N-acetyl-neuraminic acid, G is galactose, M is mannose and F is fucose.

hCG is a hormone with multiple functions during pregnancy. hCG acts on a joint hCG/LH receptor through a cyclic AMP intermediate to elicit responses. Figure 4. presents the representative structure of pregnancy hCG, a glycoprotein of molecular weight 37,180. I say representative in that variance in structures is apparent [1]. The α-subunit of hCG comprises 92 amino acids and 2 N-linked (Asn-linked) oligosaccharides [46]. The ß-subunit comprises 145 amino acids, 2 N-linked (Asn-linked) and 4 O-linked (Ser-linked) oligosaccharides [46].

hCG is measured during pregnancy by either the total hCG test, which supposedly measured all hCG variants plus its ß-subunit, or by an intact hCG assay, which supposedly measured dimeric molecules only. I say supposedly in that most laboratory total hCG tests sold today, invariably detect hCG, hCG free ß-subunit and other hCG degradation products and variants [47]. It is assumed that a similar variability occurs with intact hCG assays. I recommend one automated hCG test, the Siemens Immulite series of hCG test. This use two antibodies to the core of ß-subunit and detects most hCG degradation products and variants on a equi-molar basis [47].

Table 2 shows serum hCG concentration during pregnancy as measured by the Siemens Immulite 1000 assay. Total hCG concentration rises from pregnancy implantation (3^rd ^week of gestation) to a peak at 10 weeks of gestation. hCG levels rise exponentially during the first 7 weeks of pregnancy, increasing approximately 12-fold every week or 1.52-fold every day or 2.3-fold every two days. Total hCG concentration declines slowly from the 10 week hCG peak until a 40 week term (Table 2). hCG concentration reach 30% of peak at 15 weeks and 18% of peak at 20 weeks, and then hover close to 18% peak levels until term [48].

**Table 2:** Concentration of total hCG and hyperglycosylated hCG (hCG-H) in 496 serum samples from 310 women with term pregnancies measured using the Siemens Immulite 1000 total hCG assay

Gestation age (weeks since start of menstrual period)	N	Median Total hCG ng/ml	Range Total hCG ng/ml (variation)	Median hCG-H ng/ml	Range hCG-H ng/ml (variation)	hCG-H %
3-weeks (3 weeks 0 days - 6 days)	n = 42	0.26 (16 of 42 < 0.1 ng/ml)	0.04 - 5.5	0.20(16 of 42 < 0.1 ng/ml)	0.01 - 6.45 (645X)	87%

4 weeks	n = 42	3.4	0.21 - 173 (824X)	2.5	0.18 - 160 (888X)	51%

5 weeks	n = 67	65	1.86 - 1308 (704X)	8.6	0.96 - 698 (731X)	43%

6-weeks	n = 29	252	3.80 - 855 (225X)	86	0.76 - 629 (827X)	36%

7 weeks	n = 30	3,278	203 - 7,766 (38X)	359	27 - 931 (34X)	16%

8 weeks	n = 33	4,331	1,064 - 10,057 (9.4X)	386	67 - 1050 (15.6X)	7.0%

9 weeks	n = 24	5,832	1,031 - 11,586 (11.2X)	430	102 - 1158 (11.3X)	5.1%

10 weeks	n = 20	10,352	1,952 - 19,958 (10.2X)	521	188 - 1855 (9.9X)	4.3%

11 - 13 weeks	n = 41	5,953	1,440 - 15,318 (10.6X)	137	24 - 330 (13.7X)	2.3%

14 - 17 weeks	n = 57	2,934	311 - 4,757 (15.2X)	26	6.7 - 129 (19.3X)	1.3%

18 - 26-weeks	n = 62	1,931	210 - 6,223 (30.3X)	15.8	5.3 - 95 (17.9X)	0.65%

27 - 40 weeks	n = 49	1,911	184 - 8,530 (46.4X)	2.95	0.3 - 12.2 (40.6X)	0.14%

Data from 50 pregnancies that failed due to miscarriage were excluded from this table. Pregnancies which failed to implant in early pregnancy (total hCG < 0.1 ng/ml) are indicated in parenthesis

hCG levels vary extraordinarily widely between pregnancies [48]. hCG concentration varies among individual and among pregnancies from 0.21 ng/ml to 173 ng/ml in the 4^th ^week of gestation (variation 824X), and varies from 1.86 ng/ml to 1,308 ng/ml in the 5^th ^week of gestation (variation 704X) (Table 2). This variation can be attributed to poor dating of pregnancy, dating to the first day of the last menstrual period, rather than to the day of true start of pregnancy or implantation. The variation can also be attributed to varying hCG doubling rate among syncytiotrophoblast cells in individual pregnancies [48]. Seemingly, hCG levels rise differently among different pregnancies. As found, the spare receptor theory explains how pregnancies cope with such extreme variations in concentration. Under the spare receptor theory, when a small proportion of receptors is activated it may yield similar cellular response to when all receptors are activated [49-51]. This is due to plateaus in receptor G protein and cyclic AMP response [49-51]. Also down-regulation [52-54] can explain how a pregnancy accommodates wide variation in hCG concentration. A high concentration of hCG, for instance, may decrease the number of receptor on cells by degrading the receptor transcript rate.

hCG assays have evolved through a long history [55-60] (Table 3). The first pregnancy assay or hCG test was the famous Zondek-Aschein Test described in 1930 [55]. The Aschheim-Zondek test was based upon the observation that when urine from a female in the early months of pregnancy is injected into immature female mice, the ovaries of the mice significantly enlarge and showed follicular maturation. The test was considered reliable, with an error rate of less than 2%. This test was only replaced in 1960 with the induction of the first immunological hCG or pregnancy test, the antibody agglutination test [56] (Table 3). In this test, serum or urine was added to a tube and antibody added. When hCG was present antibody-antigen aggregates were formed. These generate a cloudy or precipitated solution.

**Table 3:** Major discoveries in hCG assays or pregnancy tests 1930-1995 [1-35,61]

Year published	Description	Authors and reference
1930	First pregnancy test, the Zondek-Aschein Pregnancy Test	Zondek B, Aschein S [55]

1960	First immunological pregnancy test, an antibody agglutination test	Wide L, Gemzell CA [56]

1967	First hCG radioimmunoassay	Aono T, Goldstein DP, Taymor ML, Dolch K [57]

1972	Discovery of hCGß radioimmunoassay, assay only detects hCG	Vaitukaitis JL, Braunstein GD, Ross GF [58]

1984	First hCG radio-immunometric assay	Armstrong EG, Ehrlich PH, Birken S, Schlatterer JP, Siris E, Hembree WE, Canfield RE [59]

1995	Automated hCG chemiluminescent-immunometric assay	Vankrieken L, Hertogh RE [60]

In 1967 the hCG radioimmunoassay replaced the agglutination test [57] (Table 3). The radioimmunoassay (RIA) was a much more sensitive and quantitative pregnancy test. In an RIA, a small but known amount of radio-iodinated hCG competed with the unknown serum or urine hCG in binding a limiting quantity of antibody. The antibody was precipitated and radioactivity measured. The lower the radioactivity the higher the unknown concentration of hCG. Unfortunately, due to the common α-subunit on hCG and LH, the hCG RIA recognized both hCG and LH. In 1972 the hCGß RIA was introduced [58] (Table 3). This was the first pregnancy test detecting only hCG.

In 1984 I saw the introduction of a new hCG antibody technology, the immunometric assay [59] (Table 3). Simply explained, an antibody to one immunological site on hCG (i.e. anti α-subunit) was immobilized on beads or on a tube. Blood or urine was added and this antibody extracted hCG, the antigen, from the solution. An antibody to a second separate site (i.e. anti ß-subunit) was labeled with a radioactivity or other tracer. This antibody, the tracer antibody, was added to the mix, it bound the immobilized antigen to form a sandwich. An immobilized antibody-hCG-tracer antibody-label complex was formed. The tracer antibody permitted quantitation of the bound antigen. The following years saw a need to automate and speed up assays, and to develop new tracers other than radioiodine. Chemiluminescence was discovered, where by a tracer emits light with limited emission of heat, as the result of a chemical reaction. In 1995 an automated chemiluminescent hCG assay was introduced [60]. Today, 2011, most laboratory hCG tests are automated and are immunometric assays using the chemiluminescent principal.

As described in Section hCG, hyperglycosylated hCG, hemochorial placentation and evolution, hCG drives uterine angiogenesis, umbilical circulation and hemochorial placentation. As described in Section hCG, hyperglycosylated hCG and pregnancy, a form of hCG seemingly drives fetal growth during pregnancy. hCG variants do as the title claims, act as a wonder molecule doing just about everything in pregnancy. Functions range from controlling uterine, fetal and placental growth during pregnancy, to protecting pregnancy from myometrial contraction, from immuno-rejection, and from macrophage rejection. All the established hCG and hyperglycosylated hCG functions during pregnancy are listed in Table 4[6,9-11,13-32,61-91].

**Table 4:** The biological functions of hCG during pregnancy

Function	References
**A. hCG**	

1. Promotion of corpus luteal progesterone production	[6,9-11,88]

2. Angiogenesis of uterine vasculature	[19-26]

3. Cytotrophoblast differentiation	[18,61]

4. Immuno-suppression and blockage of phagocytosis of invading trophoblast cells	[62-67,89]

5. Growth of uterus in line with fetal growth	[68,69]

6. Quiescence of uterine muscle contraction	[68,70-72]

7. Promotion of growth of fetal organs	[30-32,73-76]

8. Umbilical cord growth and development	[27-32]

9. Blastocysts signals uterine decidua prior to invasion regarding pending implantation	[77-80]

10. hCG in sperm and receptors found in fallopian tubes suggesting pre-pregnancy communication	[81-86]

11. hCG receptors in hippocampus and brain stem, may cause nausea and vomiting in pregnancy	[90,91]

**B. Hyperglycosylated hCG**	

1. Stimulates implantation by invasion of cytotrophoblast cells as occurs at implantation of pregnancy	[13-16]

2. Stimulates growth of placenta by promoting growth of cytotrophoblast cells	[13,14,16,17]

**C. hCG and hyperglycosylated hCG together**	

1. Drives hemochorial placentation	[87]

The original biological activity of hCG was first revealed in the 1920s [9-11]. That hCG takes over from LH in promotion of progesterone production by ovarian corpus luteal cells in pregnant women was shown in the nineteen sixties [6,88] (Table 4). As we know today, hCG only promotes progesterone production for 3-4 weeks following pregnancy implantation. This function is active for less than 10% of the length of pregnancy. As shown in Table 2, hCG reaches a peak at 10 weeks of gestation, or almost one month after progesterone promotion is complete, then continues to be produced through the length of pregnancy. Clearly, progesterone production is not the principal function of hCG even though this is the hCG function highlighted in most medical text books.

Four independent research groups show that hCG promotes an anti-macrophage inhibitory factor or a macrophage migration inhibitory factor. This is a cytokine that modulates the immune response during pregnancy. This reduces macrophage phagocytosis activity at the placenta-uterus interface, preventing destruction of the foreign fetoplacental tissue by the mothers macrophage system [62-64]. Four other groups have shown that hCG may directly suppress any immune action against the invading foreign tissue by the mother [65-67,89]. All told, hCG appears to be important in preventing rejection of fetoplacental tissue during pregnancy [62-67,89]. Most observations suggest that hCG has an inhibitory or suppressive function on macrophage activity. One group, Wan et al. [64] demonstrated that chorionic gonadotropin can directly enhance innate immunity by stimulating macrophage function.

Multiple groups have found hCG/LH receptor in the myometrium of the uterus. It has been indicated by two groups that uterine growth in line with fetal growth is controlled by hCG [68,69]. Four other groups have shown that hCG relaxes myometrial contractions during pregnancy. hCG acts on a BK-Ca calcium activated channel during pregnancy to relax the myometrium amd prevent contractions [68,70-72]. hCG levels drop during the final weeks of pregnancy. It has been suggested that this drop may be the cause of increased contractions in the weeks prior to parturition.

Four independent reports show that the blastocyst preimplantation secretes hCG into the uterine space which is taken up by hCG/LH receptors on the uterine decidual surface. In response, the decidua is prepared for impending implantation [77-80]. These non-vascular communications by hCG are a critical part of a successful pregnancy. Recent studies show the importance of hCG preimplantation signaling [81-84]. hCG signaling directly causes immunotolerance and angiogenesis at the maternal fetal interface. hCG increases the number of uterine natural killer cells that play a key role in the establishment of pregnancy [81-84].

Other new data shows other pre-pregnancy implantation function of hCG. Publications from Rao et al. and by Gawronska et al. [84-86], show the presence of an hCG/LH receptor (shown by presence of mRNA and demonstration of receptor action) in human sperm and in the fallopian tubes. The function of the hCG/LH receptor in the in sperm is unclear. It possibly has some relationship with fertility. hCG/LH receptor has recently been demonstrated in adult women's brain. CNS receptors are present in several areas of the brain such as the hippocampus, hypothalamus and brain stem [90,91]. The finding of an hCG receptor in these parts of the brain may explain why hyperemesis gravidarum or nausea and vomiting that occurs during normal pregnancy.

Exciting new research by multiple research groups is finding hCG/LH receptors in fetal organs. Goldsmith et al. [73], have found hCG/LH receptors in the fetal kidney and liver. Rao et al. [30-32,74,75], have located hCG/LH receptors in fetal lungs, liver, kidneys, spleen and small and large intestines. Interestingly, this hCG/LH receptor is present in the fetal organs but completely absent in the adult organs. Seemingly, hCG/LH receptors disappear at birth.

It is suggested that hCG may promote organ growth and differentiation in the fetus. The human fetus seemingly produces its own hCG from the fetal kidneys and liver [73,76]. The concentrations in fetal circulation are much lower than maternal concentrations, suggesting that placental hCG secretion is directed towards the maternal circulation only and is prevented from entering into fetal circulation [76]. While the hCG receptor has been shown in fetal organs, no function has been directly demonstrated, just suggested. As such, all the findings regarding the fetus have to be considered as just suggestions at this time. Unfortunately, most animals do not make a form of hCG, making the role of hCG in the fetus difficult to prove.

This review claims that there are 5 hCG variants with independent biological activity [44]. There may actually 6 independent hCG variants when one considers fetal hCG. It is made by fetal kidneys and liver [73,76] so may not be structurally be similar to syncytiotrophoblast sialylated hCG. Our supplies of fetal hCG may be limited to umbilical cord hormone. I am not sure that sufficient fetal hCG will ever be collected to permit structural analysis. Generally speaking only placental and pituitary cells can make glycosylated hCG dimer, other cells only produce free subunits. I wonder, is fetal hCG a dimer, and normally glycosylated? It is only an hCG/LH receptor that has been found in the fetus. Does the fetus produce hyperglycosylated hCG as a growth promoter, acting on a TGFß receptor?

**Figure 5:**
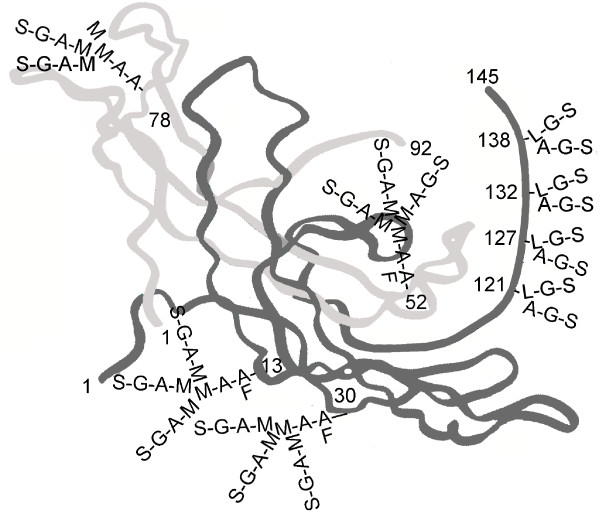
**The three dimensional structure of hyperglycosylated hCG **[4]. In order to form crystals hCG was first deglycosylated. hCG and hyperglycosylated hCG have identical amino acid sequence [1], yet vary in function. hCG binds the hCG/LH receptor while hyperglycosylated hCG antagonizes a TGFß receptor [6-8]. As such the glycosylation (all removed) has to make a structural difference to the two hCG forms. This difference is not shown. Oligosaccharide are added to the three dimensional structure assuming the carbohydrate structure observation of Elliott et al. [1]. Oligosaccharides are charged so must project towards the surface of the molecule. L in oligosaccharides is N-acetylgalactosamine, A is N-acetylglucosamine, S is sialic acid or N-acetyl-neuraminic acid, G is galactose, M is mannose and F is fucose.

Hyperglycosylated hCG is a super-glycosylated variant of hCG. While hCG is made by fused placenta syncytiotrophoblast cells, hyperglycosylated hCG is made by root placental cytotrophoblast cells (13,46). As shown in Figure 5 hyperglycosylated hCG is a variant of hCG with double-size O-linked oligosaccharides and triantennary (vs. biantennary) N-linked oligosaccharides. Hyperglycosylated hCG shares all 92 amino acid α-subunit and a 145 amino acid ß-subunit with hCG. Hyperglycosylated hCG is an extreme molecule, molecular weight 42,800, 39% sugar by molecular weight. Hyperglycosylated hCG is the most acidic glycoprotein known to humans, the peak acidity is pI 3.1.

Table 2 shows the concentration of hyperglycosylated hCG in human serum during pregnancy. As shown, hyperglycosylated hCG is the principal hCG form produced in early pregnancy. Hyperglycosylated hCG accounts for 87% of total hCG in the 3 rd week of gestation and 51% of total hCG during the 4^th ^week of gestation, the proportion hyperglycosylated hCG rapidly declines after this point. The high hyperglycosylated hCG in early pregnancy is thought to be the driving signal of deep pregnancy implantation. Not all commercial laboratory, research, point-of-care and home pregnancy tests detect hyperglycosylated hCG equally with hCG. This may make a test inappropriate for early pregnancy detection [47]. In Laurence Cole PhD experience only the Siemens Immulite series of tests is appropriate for laboratory tests, only the Quidel and Beckman series of tests are appropriate as Point-of-Care pregnancy tests and only the Church and Dwight First Response and Answer Home tests are appropriate at Over-the-Counter pregnancy tests. Hyperglycosylated hCG can be separately detected to total hCG using the antibody B152 immunometric test, which only tested hyperglycosylated hCG [92].

Hyperglycosylated hCG and hCG free ß-subunit are interchangeable cancer promoters functioning by antagonizing a TGFß receptor [7,8]. It is thought that hyperglycosylated hCG produced in early pregnancy promotes implantation by a similar mechanism, involving antagonism of a TGFß receptor, blockage of apoptosis, and promotion of metalloproteinase and collagenase production [93-95].

Hyperglycosylated hCG has multiple clear functions in human pregnancy implantation and promotion of cytotrophoblast growth (Table 4). Hyperglycosylated hCG is demanded by pregnancy with it unique properties as a TGFß antagonist to drive pregnancy implantation. Implantation is driven in a cancer-like manner. In humans implantation passed through the uterine decidua, uterine stroma and connectictive tissue into the uterine myometrium or muscle surrounding the uterine endometrium. Placenta normally implants at 1/3^rd ^the depth or the myometrium or approximately 40% of the depth of the uterus. Implantation seemingly uses the hyperglycosylated hCG-induced cytotrophoblastic metalloproteinases and collagenase activities to achieve its goal [94,95]. Cytotrophoblast hyperglycosylated hCG as an autocrine also promotes root placental cytotrophoblast growth during the course of pregnancy to develop and prime growing villous placental tissue (13,14,16,17).

### Hyperglycosylated hCG, failing pregnancy and hypertense pregnancy

As discussed in Section hCG, Hyperglycosylated hCG, Hemochorial Placentation and Evolution, and Section hCG, Hyperglycosylated hCG and pregnancy, human hyperglycosylated hCG is the extreme end product of evolution that drives pregnancy implantation to it extreme, and drives production of hemochorial placentation to the extreme. Humans having an extreme molecule to drive implantation are faced with the intricacies and demands of an extreme molecule, so have to face a high proportion of rejected pregnancies, this is miscarriage or spontaneous abortions (17% failure rate) and biochemical pregnancies (25% failure rate) or 25% + 17% or 42% pregnancy failure rate [15,96]. Simian primates only have an 8% failure rate since they do not have to cope with human hyperglycosylated hCG.

Scientist show that most biochemical pregnancies and spontaneous abortion pregnancies, approximately two-thirds, are due incomplete implantation of pregnancy [97,98]. As shown by us in two studies examining 62 pregnancies and 127 pregnancies [15,99], all (100%) of normal term pregnancies (81 and 42 total) produced greater than 40% hyperglycosylated hCG (% of total hCG) on the day of implantation. In contrast, only 8 of 36 and 7 of 20 biochemical and spontaneous aborting pregnancies produced greater than 40% hyperglycosylated hCG on the day of implantation. It is assumed that the failures exceeding 40% hyperglycosylated hCG are the rare genetic abnormalities and that the bulk, 28 of 36 and 13 of 20 pregnancies are pregnancy failures due to improper implantation. It is concluded that hyperglycosylated hCG is an absolute marker of biochemical pregnancy and spontaneous abortion, and that deficiency of hyperglycosylated hCG (< 50% hyperglycosylated hCG) is the actual cause of human pregnancy failures [15,99].

Unfortunately, to achieve the absolute differentiation of term pregnancy and failing pregnancy the testing has to be performed on the day of implantation of pregnancy. Detection of approximately 80% term pregnancies is possible at later weeks of pregnancy [92,100].

The hyperglycosylated hCG test is licensed to Quest Diagnostics Inc., and can be ordered from any world-wide Quest Diagnostics laboratory. It also can be ordered from the USA hCG Reference Service. The hyperglycosylated hCG test is also valuable as a high sensitivity marker for Down Syndrome screening [101,102]. This screening service is currently offered by Quest Diagnostics Inc.

As published, The future sees prenatal screening being expanded to pregnancy induced hypertension (PIH) and preeclampsia screening, using a hyperglycosylated hCG test. A study by Bahado-Singh and colleagues [103] and more recent confirming studies by Brennan and colleagues (papers in preparation) shows that hyperglycosylated hCG measurements may be invaluable for screening women in the first and second trimester of pregnancy for these two complication of pregnancy. Today, preeclampsia is the biggest cause of maternal death in pregnancy. Hyperglycosylated hCG promotes hemochorial placentation formation [87], preeclampsia and PIH are complication of ineffective hemochorial placentation, thus it is marked by unduly low hyperglycosylated hCG. New hyperglycosylated hCG prenatal screening tests will identify women at high risk for PIH and preeclampsia.

### Sulfated hCG and the menstrual cycle

Sulfated hCG produced by the pituitary is barely detectable during the menstrual cycle. Sulfated hCG parallels LH during the menstrual cycle. In a recent study, at the time of the LH peak, hCG level in 277 menstrual cycles averaged 1.54 ± 0.90 mIU/ml [104]. In general, during the menstrual cycle sulfated hCG levels are low, approximately one fiftieth of circulating LH level [104-106]. While these levels are small, sulfated hCG is exactly 50-fold more potent that LH [5]. As such sulfated hCG may perform a comparable job to LH in promoting androstenedione production during the follicular phase of the menstrual cycle, a comparable job in promoting ovulation and corpus luteal formation. It may also perform a comparable job to LH in promoting progesterone production in the luteal phase of the menstrual cycle. All told, one can no longer correctly say that LH promotes ovulation, it is LH plus sulfated hCG [5,104-106].

During the menstrual cycle, hypothalamic gonadotropin releasing hormone (GnRH) pulses stimulate follicle stimulating hormone (FSH) ß-subunit and LH ß-subunit genes (the a-subunit is produced in excess) in pituitary gonadotrope cells. The problem is that on chromosome 19 there is a single LH ß-subunit gene located next to 8 hCG duplicated ß-subunit genes. GnRH bombards LH ß-subunit gene and cannot help bombarding some of the adjacent hCG ß-subunit genes leading to pituitary hCG production. Gonadotrope cells can sulfate glycoproteins leading to partially sulfated hCG, LH and FSH.

In menopause, with the absence of steroid feedback to the hypothalamus, GnRH pulse become maximal. The result is promotion of vast excesses of LH, hCG and FSH are produce by gonadotrope cell due to excessive GnRH pulses. Serum LH increases from, 1-90 mIU/ml to > 100 mIU/ml in menopause, serum FSH increases from 1-29 IU/L to 30-200 IU/L in menopause, and serum hCG from < 1 - 3 mIU/ml to 2-39 mIU/ml in menopause. All told, pituitary sulfated hCG is very detectable in a menopausal woman [104-108]. Perimenopause is the stage prior to menopause marked by oligomenorrhea or irregular menstrual periods. Just as perimenopause is marked by the start of raised LH and FSH production, it is also marked by detectable hCG levels.

Exceptional women can achieve pregnancy up to 60 years age. In general, women 40-55 years age are likely to be in perimenopause, and those > 55 years age are in menopause [107]. As reported, the range of hCG detection in non-pregnant menopausal women, > 55 years age, is < 2 to 13.1 mIU/ml, in non-pregnant menstrual women 18-40 years age is < 2 to 4.6 mIU/ml, and potential perimenopause women is < 2 to 7.7 mIU/ml [107]. In the USA hCG Reference Service experience, hCG levels as high as 29 mIU/ml, median 7.2 mIU/ml have been detected in perimenopause and as high as 33 mIU/ml, median 8.0 mIU/ml are detected in menopause (Table 5). Higher hCG levels have been recorded, as high as 39 mIU/ml, in women having oophorectomy (Table 5).

**Table 5:** Use of serum free ß-subunit (hCGß plus hyperglycosylated hCGß) and urine ß-core fragment as tumor markers for detection of malignancies

Malignancy	hCGß as a tumor marker		ß-core fragment as a tumor marker	
	
	Number of Cases	Serum hCGß (> 3 pmol/L)	Number of Cases	Urine ß-core fragment (> 3 pmol/L)
Bladder cancer	170	35%	102	48%

Cervical cancer	60	37%,	410	48%

Colorectal cancer	436	17%		

Endometrial cancer	55	33%	157	47%

Lung cancer	243	18%	122	45%

Ovarian cancer	150	38%	207	66%

Pancreatic cancer			29	55%

Vulvar	64	41%		

TOTAL	1164	Mean 30%	1027	Mean 48%

All averages are determined by combining total positive cases from multiple reports [109-130,132-138]

The USA hCG Reference Service has examined 88 women producing pituitary sulfated hCG. This is a list of only referred cases and is not a random list of woman over 40 years old. Among the 88 cases, sulfated hCG ranged from 1.8 mIU/ml to 39 mIU/ml [106]. In menopausal cases the median level was 8.0 mIU/ml, in perimenopausal cases the median was 7.2 mIU/ml and in induced menopausal cases, women receiving oophorectomy, the median was 6.3 mIU/ml.

Research by Gronowowski et al. [108], shows that measurement of FSH levels is a powerful predictor of pituitary sulfated hCG (FSH > 30 IU/L). The USA hCG Reference Service started using FSH as a confirmation of pituitary sulfated hCG one year ago. We both confirm and support the use of FSH testing to affirm the diagnosis of pituitary hCG. Once a woman is diagnosed as producing pituitary sulfated hCG, what do you do next? The only answer is "nothing," it is normal, it is natural, you need to completely ignore it. A physician can confirm that we are dealing with pituitary hCG, as described above, by administering a high estrogen oral contraceptive, to suppress hCG, for 3 weeks.

### Hyperglycosylated hCG, hCG free ß-subunit and cancer

My PhD at Medical College of Wisconsin was regarding structure of this hCG-variant produced by 2 cervical cancer cell lines, DoT and Caski. As shown, these cell lines produce a invariably glycosylated variant of hCG free ß-subunit. Soon after getting my PhD, I went on to a postdoctoral fellowship at University of Michigan in Ann Arbor and showed that hCG free ß-subunit was a tumor marker present in culture fluids, serum and urine, and patented hCG free ß-subunit and its variants including ß-core fragment as a tumor markers [109]. This patent was licensed by Ciba-Corning Laboratories, the predecessor to Quest Diagnostics 1985-1999,

Multiple studies in the early 1980s showed that serum hCG free ß-subunit and urine ß-core fragment its degradation product, were tumor marker for all cancers (Table 5). As shown and averaged over multiple studies [109-125] (Table 5), hCG free ß-subunit and ß-core fragment marked a proportion of all malignancies. All told, hCG free ß-subunit marked 30% of 1,164 malignancies in serum tests, at the extremes, 17% of colorectal cancers, and 38% of ovarian cancers [109-125] (Table 5). All told and averaged, hCG ß-core fragment marked 48% of 1,027 malignancies in urine tests, at the extremes, 45% of lung cancers, and 66% of ovarian cancers (Table 5) [109-125].

In the years that followed Acevedo and Krichevsky [126] and Regelson [127] used cancer membrane flow cytometry methods to show that all advanced cancers produced an hCG free ß-subunit variant. An investigation into hCG degradation at that time showed that hCG free ß-subunit (circulating 1/2-life 0.72 h) is removed from the circulation much more rapidly than hCG (circulating 1/2-life 36 h) [128]. Research showed that hCG free ß-subunit is nicked upon entering the circulation, and possibly loses its C-terminal peptide [129,130]. As such hCG free ß-subunit had a circulating 1/2-life of just seconds, seconds before it is degraded and excreted in the kidney, or removed by the liver. With the miniscule circulating survival time, the claim that all malignancies produced hCG free ß-subunit, while it could only be detected in the serum in just 30% of cases made sense. Clearly, most of the hCG free ß-subunit produced by cancers was cleared rapidly and was undetectable as a tumor marker.

Research into choriocarcinoma showed that this cancer produced hyperglycosylated hCG [1,131]. It was not until the mid-2000s that it was shown that hyperglycosylated hCG was a separate and independent molecule to hCG acting on cytotrophoblast cells in pregnancy implantation, and in choriocarcinoma cells [13,14]. As demonstrated, hyperglycosylated hCG directly promoted choriocarcinoma cytotrophoblast cell growth (in cell culture) and invasion (in Matrigel chambers). Regular hCG had no effect [13,14]. When choriocarcinoma cells were transplanted into nude mice cancer grew rapidly. Cancer cell growth and malignancy could be totally suppressed, oncostasis, by treating the nude mice with hyperglycosylated hCG antibody [13,14]. Clearly, hyperglycosylated hCG was the single driving signal of choriocarcinoma. It was then shown that ovarian and testicular germ cell malignancies took on choriocarcinoma-like cytotrophoblast morphology, producing high concentrations of hyperglycosylated hCG, like choriocarcinoma, and driven by hyperglycosylated hCG like choriocarcinoma cells [14].

While research with hyperglycosylated hCG and choriocarcinoma was ongoing in the USA, research in Europe was continuing to investigate hCG free ß-subunit and other malignancies. As found in the 1990s, the detection of hCG free ß-subunit in serum was a marker of poor prognosis of cancer [132]. Later, it was shown that hCG free ß-subunit secreted by cancer cells directly stimulated cancer cell growth [7,133-138]. hCG free ß-subunit blocked apoptosis in cancer cells and enhanced growth and malignancy [7,133-138]. In 2000, Stephen Butler PhD in Europe showed that hCG free ß-subunit produced by bladder cancer cells bound and antagonized a TGFß receptor on cancer cells [7].

Recently, I examined hyperglycosylated hCG and 2 choriocarcinoma cell lines, and hCG free ß-subunit and 2 bladder cancer lines and 2 endometrial cancer cell lines [8]. I confirmed, hyperglycosylated hCG promoted growth of both choriocarcinoma cell lines and hCG free ß-subunit promoted growth of both bladder and endometrial cancer cell lines [8]. Intriguingly, they were interchangeable and could take each other's roles, hyperglycosylated hCG could promote bladder and endometrial cancer, and hCG free ß-subunit could promote choriocarcinoma [8]. It was rapidly inferred that both antagonize a TGFß receptor making them interchangeable.

This research seemingly tied together our knowledge regarding hCG and cancer, interlinking the general cancer stories of Europe and the choriocarcinoma amd germ cell cancer stories of the USA. The story of hCG and cancer was very much enhanced and confirmed by ongoing clinical trials with hCG vaccines and advanced malignancies. Three companies, Celldex, CG Therapeutics and MCI BioPharma Inc. started in 2000 testing a synthetic hCGß vaccine in treating advanced cancer cases [139-144]. Results were very exciting, with the finding that hCGß vaccines are considerably extending lives of advanced cancer patients. For example, examining the clinical trial with hCGß vaccine and colorectal cancers [141], the average survival of those with optimal antibody response was 45 weeks, compared to just 24 weeks in those without optimal response (p = 0.0003). This demonstrated that hCG antibodies could potentially double longevity. Similar results have been reported with prostate cancer, lung cancer and breast cancer.

**Figure 6:**
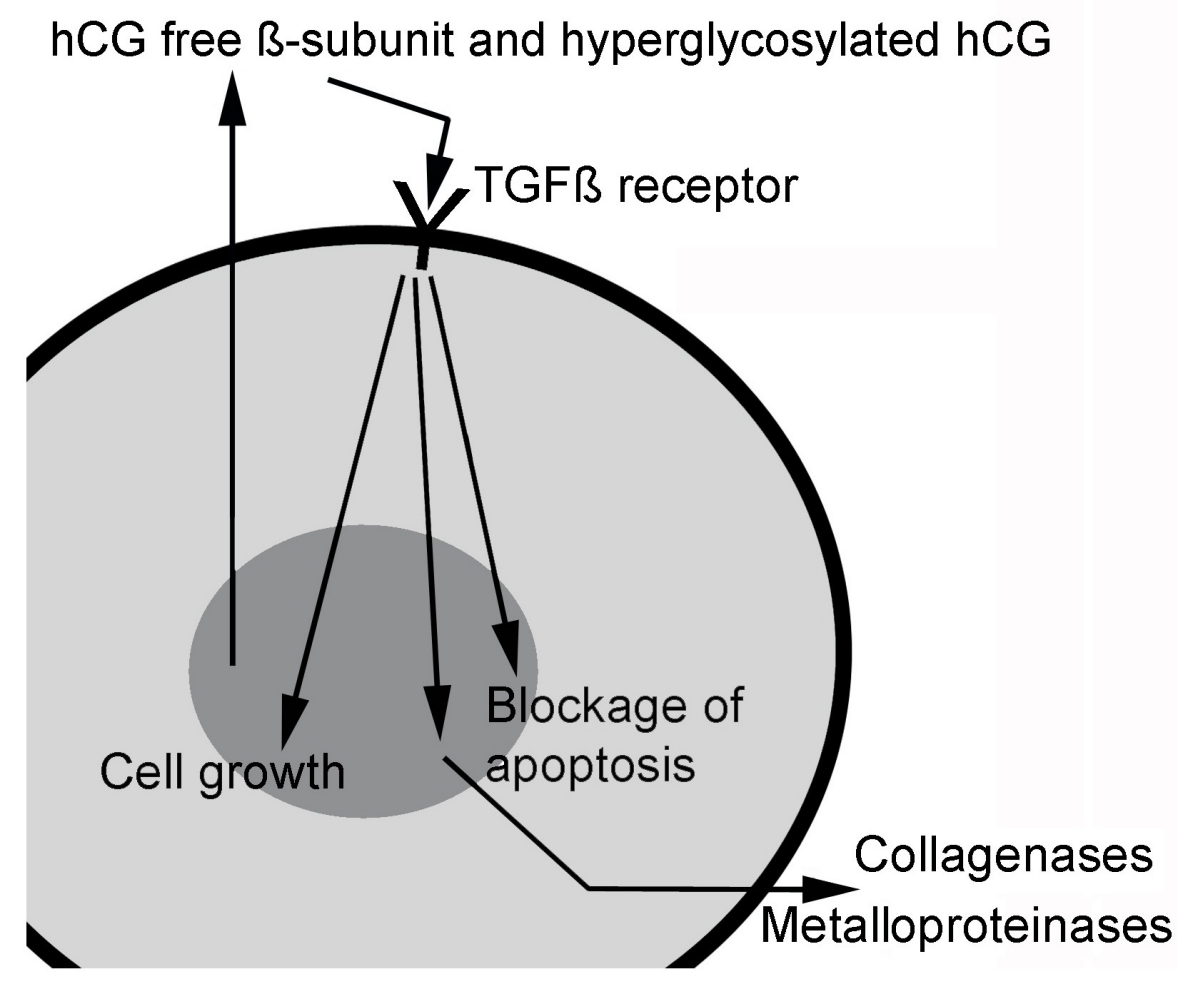
**Proposed pathways of free ß-subunit and hyperglycosylated hCG in advanced cancer cases**. As illustrated, free ß-subunit and hyperglycosylated hCG are hCG variants with exposed TGFß binding structures [1-4,6], these are autocrines which antagonize the TGFß receptor, promoting cell growth and blocking cell apoptosis. As a result of the antagonism, collagenases and metalloproteinases are produced by cells [94,95]. As illustrated, cells secrete hCG free ß-subunit or hyperglycosylated hCG. This enters the circulation and rotates around the body. hCG free ß-subunit of hyperglycosylated hCG then bind back on a receptor on the cancer cells, a TGFß receptor, and antagonize this receptor. As a result cell growth is promoted, and cell apoptosis is blocked. Cell secrete collagenases and metalloproteinases.

In conclusion, there are seemingly 2 kinds of cancer as relates to hCG variants. Type 1 is a cancer of hyperglycosylated hCG producing cells, choriocarcinoma, gestational trophoblastic neoplasm, and ovarian and testicular germ cell cancers. This type of cancer produces hyperglycosylated hCG from the start of malignancy. Hyperglycosylated hCG seemingly work through a mechanism involving antagonism of TGFß (proposed mechanism, Figure 6) [7,8]. This type of cancer is seemingly modulated completely by hyperglycosylated hCG, its growth, metastases and grade.

The second type of cancer was seeming represented by all other human malignancies, include lung cancer, breast cancer, leukemia, lymphoma and so on. These cancers start out as transformed cell driven by an hCG-independent process. As the cancer progresses and becomes advanced it is able to express the hCG ß-subunit gene and make hCG free ß-subunit. The hCG free ß-subunit driven TGFß antagonism mechanism (proposed mechanism, Figure 6) takes over control of the cancer, as indicated by the vaccine studies, and has complete control of the advanced disease. Based on the vaccine studies, development of a human high affinity hCG ß-subunit antibody may be a future answer to human cancer treatment. Vaccine only works in people with a good functioning immune system. This is where hCG ß-subunit antibody may shine. I believe that hCG ß-subunit antibody based on nude mouse experiments [13,14], could someday cure Type 1 cancers, and could seemingly offers greatly improved treatment and improved longevity to all Type 2 malignancies.

## Conclusions

hCG is a wonder of today's science. It firstly is extreme molecule, including features such as the most acidic molecule, the most glycosylated molecule and the longest circulating half life. It secondly is unique with multiple variants of hCG having independent functions, and hCG variants binding 2 separate receptors, hLG/LH receptor and TGFß receptor. hCG needs to be considered as a placental hormone and autocrine, a pituitary hormone and a major cancer promoter.

## Competing interests

The author has no conflict of interest in writing this paper, other than being the Director of the USA hCG Reference Service. The author does consult for Church and Dwight Inc on home pregnancy tests, for Siemens Diagnostics Inc. on hCG pregnancy tests, and for Quest Diagnostic Inc. on hyperglycosylated hCG. This consulting has had no influence in this research.

## Author's information

Laurence A. Cole PhD, The Howard and Friedman Distinguished Professor of Obstetrics and Gynecology, USA hCG Reference Service, 2412 Calle De Panza NW, Albuquerque NM 87104, Phone: 505-263-9635, E-mail: larry@hcglab.com.
